# Supplementary material for: Ongoing Strategies to Improve Antimicrobial Utilization in Hospitals across the Middle East and North Africa (MENA): Findings and Implications
Source: Antibiotics (Basel). 2023 Apr 28;12(5):827. doi: 10.3390/antibiotics12050827 (PMC10215537; doi:10.3390/antibiotics12050827)
Supplement: Supplementary file 1 [file antibiotics-12-00827-s001.zip › antibiotics-2313717-supplementary.pdf]

Table S1. – Concerns with the development of SSIs among MENA Countries and potential risk factors.

Country	Author and year	Study background and aims	Findings and implications
**Middle Income Countries***			
Jordan	Jalil et. al, 2017 [191]	• There were concerns with the high rate of SSIs following caesarean sections<br>• Determine possible risk factors to reduce SSIs in the future	Risk factors influencing SSIs included:<br>• Body mass index ≥36 kg/m2 prior to pregnancy<br>• Hospital stay longer than 3.5 days<br>• Having the operation at a gestational age greater than 40 weeks<br><br>• Prescribed a higher weight-adjusted dose of cefazolin prophylactically for SAP was associated with lower odds of an SSI
Jordan	Ennab et. al 2022 [17]	Identify bacteria from SSIs to determine antimicrobial susceptibility profiles to guide future antibiotic choices	• 17 out of the 28 identified bacteria had resistance levels above 0.2 including *Escherichia coli*, *Pseudomonas aeruginosa*, *Proteus mirabilis*, *Klebsiella pneumoniae*, *Staphylococcus aureus* and *Streptococcus pyogenes*<br>• Overall, hospital treatment protocols should be enforced and monitored to reduce current resistance rates
**High-Income Countries***			
Kuwait	Hamza et. al, 2018 [192]	• Assess the prevalence of SSIs following gastrointestinal procedures<br>• Determine possible risk factors that may be present to improve future patient management	• Following gastric, colon, and small bowel 1operations, respectively, 0.8% (13/1722), 19.8% (38/192), and 10.8% (20/185) of 2,099 patients experienced SSIs<br>• The predominant pathogens causing SSIs were gram-negative bacilli (60% multidrug-resistant organisms) with a significant prevalence of multidrug-resistant pathogens<br>• In stomach procedures, using an endoscope showed a protective effect against SSIs<br>• Implementing focused preventative measures for identified risk factors should reduce SSIs
Saudi Arabia	Alkaaki et. al. 2018 [61]	Describe the incidence, bacteriology, and risk factors related to SSIs	• 16.3% of patients (55/337) had documented SSIs – 5 had deep infections (9% - 5/55) and 25 (45% - 25/55) had both superficial and deep infections<br>• The incidence of SSIs was 35% versus 4% in open versus laparoscopic procedures respectively (p 0.001)<br>• *Escherichia coli* (26 patients – 52%) and gram positive bacteria (19 patients – 38%) were the most typical bacteria to be isolated<br>• In patients susceptible to SSIs, antibiotic preventive regimens need to be tailored for maximum impact
Saudi Arabia	Alsaeed et al., 2022 [60]	• Out of 209 surgical patients, 52 patients did not receive preoperative antibiotics (control)<br>• 157 received SAP	• One patient in the prophylactic group developed an SSI (0.6%) versus three patients in the control group (5.8%), which was statistically significant<br>• The mean hospital length of stay in the group receiving prophylaxis at 38.5±9.2 hours was significantly shorter than the control group at 57.3±12.1 hours<br>• The most commonly prescribed antibiotics for SAP were metronidazole (**A**), cefuroxime (**W**), cefazolin (**A**), and ceftriaxone (**W**)<br>• Prescribing preoperative antibiotics significantly reduced SSIs and the mean length of hospital stay

United Arab Emirates	Alnajjar et al., 2020 [62]	Identify key elements associated with SSIs following a caesarean section, with 100% of mothers receiving a single dose of cefazolin within one hour of skin incision	• SSIs occurred in 1.4% of patients following a caesarean section<br>• Increased gestational age of the fetus was a reliable predictor of SSIs<br>• Targeted health care policies should be informed by identified risk factors to lower the incidence of SSIs
United Arab Emirates	Alshehhi et al., 2021 [67]	• Assess the appropriateness of antibiotic administration to prevent SSIs, particularly the length of administration<br>• Ideally, the maximum length should be up to 24 hours post-operatively	• Antibiotics for SAP were typically administered for 3 days (104 patients; 32.9%); 5 days (89 patients; 25.7%) and 7 days (41 patients; 11.8%) prior to an ASP<br>• The average duration of cefuroxime (most prescribed antibiotic – **W**) pre-ASP was 3.8 ± 1.2 days

NB: ASP: Antimicrobial Stewardship Program; AWaRe classification for antibiotics – A = Access, W = Watch, R = Reserve [45]; SAP: Surgical Antibiotic Prophylaxis; SSIs: Surgical Site Infections; * World Bank Status (Based on [131]).
